# Fabrication of Soft-Oxometalates {Mo_132_} Clusters With Novel Azobenzene Surfactants: Size Control by Micelles and Light

**DOI:** 10.3389/fchem.2021.625077

**Published:** 2021-02-12

**Authors:** Zhe Wang, Xuefeng Li, Shengli Chen, Jinfeng Dong

**Affiliations:** Engineering Research Center of Organosilicon Compounds and Materials, Ministry of Education, College of Chemistry and Molecular Sciences, Wuhan University, Wuhan, China

**Keywords:** soft-oxometalates, azobenzene surfactants, solubilization, light response, redox potential

## Abstract

Soft-oxometalates (SOMs) are colloid suspensions of superstructured assemblies of polyoxometalates (POMs) and are found to be very effective photo-catalysts in a number of chemical reactions. The stabilization of SOMs generally requires legends or stabilizers, e.g., polymers and surfactants. In this paper, a light responsive azobenzene surfactant, C_10_AZOC_2_N_3_, was developed and used to stable {Mo_132_} SOMs. Various techniques such as Dynamic light scattering, TEM, UV-Vis spectra and cyclic voltammetry were employed to characterize the experimental results. The outstanding structure-directing effect of surfactant self-assembly micelles in solution on inorganic counter-anions was demonstrated. Different amount of cyclohexane was solubilized into C_10_AZOC_2_N_3_ micelles to successfully control the size of {Mo_132_} SOMs cluster. Furthermore, the clusters exposed to UV light for a certain time can be served as a second trigger to control the size of SOMs due to the *trans*-cis conformation transition of surfactant molecules. The redox potentials of C_10_AZOC_2_N_3_-{Mo_132_} SOMs were investigated as the cluster size varied. Interestingly, the redox potential of {Mo_132_} was not affected by the cluster size, indicating that the presence of surfactant did not change the main function of {Mo_132_} as an electrochemical catalyst, but merely assisted in the size control of SOM aggregation.

## Introduction

Polyoxometalates (POMs) are nano-sized inorganic transition metal-oxide based molecular clusters, attracting wide attention due to their structural, catalytic, electrochemical and biological properties (Miras et al., 2012; Wang and Yang, 2015). Since the early 1990s when Pope and Müller (Pope and Müller, 1991) revealed the interesting features and the potential of these unique metal-oxide clusters, polyoxometalates research field has developed rapidly. POMs have been widely used in the construction of nano-systems and functional materials for photo- and/or electro-catalysis and biomedicine in recent years (Anyushin et al., 2020). For example, Hill et al. (Lv et al., 2014) synthesized a carbon-free, oxidatively and thermally stable, homogeneous water oxidation catalyst based on redox-active (vanadate(V)-centered) polyoxometalates ligands to catalyze water oxidation in dark and visible-light-driven conditions, exhibiting higher hydrolytic stability, exceptionally fast catalysis under mild conditions and higher selectivity for water oxidation vs. bpy ligand oxidation. Shi et al. (Zhang et al., 2016) achieved the acidity/reducibility-specific photothermal conversion using a Mo-based polyoxometalates cluster with self-adaptive electronic structure, providing a promising clinical photothermal agent and expected to establish a new physicochemical paradigm for smart theranostic medicine design based on POM cluster. Very recently, it has been reported that POM molecules can self-assemble into supramolecular assemblies in solution which was named soft-oxometalates (SOMs) (Roy, 2014; Crans and Roy, 2016). SOMs take the advantages of POM fundamental properties, soft matter properties and biological effects, and are found to be very effective photo-catalysts. For example, Soumayajit Roy et al. (Das et al., 2016) fabricated a soft-oxometalate-based photocatalytic system that readily oxidizes water to oxygen, and then they further used the SOM-based catalytic system to photocatalytic reduction of carbon dioxide (Barman et al., 2018; Biswas et al., 2018), providing a new system for catalysis. In nanocatalysis, the activity of a catalyst has been shown to be inversely proportional to the surface area (Mizuno and Misono, 1998). Hence, a directed effort in catalyst design goes toward controlled design of the catalyst where the catalyst size is engineered. However, these SOMs self-assembled by POMs are often random at most in the range of a few nanometers (Liu, 2010). Controlling the size and morphology of POMs at the mesoscale is still a formidable challenge (Roy, 2014). Thus, fabricating the ordered supramolecular SOM catalysts in a controlled manner is needed.

There are numerous techniques to fabricate ordered supramolecular structure, among them, molecular aggregates constructed by surfactant self-assembly, including spherical micelles, rod-like micelles and vesicles, have been established and understood well (Song et al., 2014). The morphology of these surfactant aggregates can be manipulated by varying various parameters, e.g., concentration, solvent polarity, temperature, pH, electrolyte and additives (Chu et al., 2013; Jiang et al., 2015). Since those aggregates are ordered according to their molecular polarity, non-polar additives can be solubilized and enlarge the size of the aggregates. Hence, surfactants are also one of the most commonly used templates to induce the synthesis of advanced ordered micro-nanostructures. To support or induce inorganic materials to form ordered supramolecular aggregates, non-covalent interactions such as electrostatic interaction or hydrophobic interaction between surfactant molecules and inorganic anions are the most common driving force (Huang et al., 2016; Bryant et al., 2017). When POMs act as chaotropic or salting-in anions in surfactant solutions, combination of POMs and opposite charged cationic surfactants can result in various self-assembly structures, such as vesicles, surfactant encapsulated complex and lyotropic liquid crystals (Fan and Hao, 2009; Floquet et al., 2012; Li et al., 2017). In that case, when the surfactant micelles act as support templates, the surfactant-POM self-assembly structures may be regulated by varying the shape and size of micelles, whereas environmental stimulation, such as temperature, pH, light and magnetism, may play some roles. Here we propose to employ a photo-responsive surfactant to fabricate the morphology of SOMs.

Photo-responsive surfactants have long been known as the most successive functional surfactants due to their noninvasive stimuli response. On one hand, the surface property of those surfactants itself can be switched on and off depending on the resource of light (Pernpeintner et al., 2017). On the other hand, many materials can be fabricated as functional applications due to the *cis*- or *trans*-conformation of surfactant molecules (Santer, 2018). For example, Chen et al. (Ren et al., 2019) fabricated a photo-responsive system where photo-responsive azobenzene units within the amphiphilic precatalysts allowed for switching between assembled and disassembled states, thereby modulating the catalytic activity which reliant on intermolecular cooperative effects when amphiphiles assemble into vesicular structure. In this work, a photo-sensitive cationic surfactant micelle bearing an azobenzene group in its hydrophobic chain is going to be used as templates to design SOMs with an anionic {Mo_132_} Keplerate as oxometallates, aiming to understand the shape control of SOMs by the said surfactant.

## Experimental Section

### Materials

All the materials and reagents were purchased from commercially available sources and used as received. The azobenzene surfactant, 1-[2-(4-decylphenylazo-phenoxy)-ethyl]-1-di-ethylenediamine (C_10_AZOC_2_N_3_), was homemade by following the previous work (Wang et al., 2019). The detailed synthetic route of C_10_AZOC_2_N_3_ was shown in Supplementary Scheme S1. Ultrapure water (18.2 MΩ cm at 25°C) was made by Millipore-Q.

### Synthesis of (NH_4_)_42_[Mo^VI^_72_Mo^V^_60_ O_372_(CH_3_COOH)_30_(H_2_O)_72_]·hydrate

{Mo_132_} was synthesized by following a literature procedure (Ren et al., 2019). 5.6 g (4.5 mmol) (NH_4_)_6_Mo_7_O_24_·4H_2_O and 12.5 g (162.2 mmol) CH_3_COONH_4_ were dissolved in 250 ml water, and then 0.8 g (6.1 mmol) N_2_H_4_·H_2_SO_4_ was added. After stirring for 10 min, the solution turned blue-green. Slowly add 83 ml 50% (v/v) CH_3_COOH solution and stir for 5 min until the color turns green. The green solution was standing in an open vessel and turned brown gradually. After 4 days, the dark brown mixture was filtrated under vacuum. The dark brown crystals were washed with 90% ethanol first and then with mixture solvents of ethanol and ethyl ether 2–3 times, and dried under vacuum. The yield of {Mo_132_} is 3.0 g.

### Preparation of C_10_AZOC_2_N_3_-{Mo_132_} Soft-Oxometalates

Firstly, a set of surfactant micelle solutions of five sizes was prepared. To do so, different volumes (0, 10, 20, 30, 40 μL) of cyclohexane were added to 10 ml 0.1 mmol/L C_10_AZOC_2_N_3_ aqueous solution and dispersed uniformly by ultrasound. Then, a set of five corresponding dispersions of SOMs was prepared by adding different volumes (10–200 μL) of 1 mmol/L {Mo_132_}, mixing evenly by stirring for 5 min. These SOM dispersions were balanced in the dark for at least 12 h, and were used for characterization and further experiments.

### Light Irradiation

Samples were placed in a glass cuvette and irradiated under UV light for 10–30 min at a distance of 15 cm. A flashlight LED UV lamp (OPX-365, λ = 365 nm, 15 mW/cm^2^) was employed as the UV light resource.

### Characterization

Dynamic light scattering (DLS) and zeta potential measurements were conducted both using a Malvern ZEN 3600 Zetasizer instrument. All the measurements were carried out three times at 25°C and the average values were reported. Transmission electron microscopy (TEM) and energy dispersive X-ray spectroscopy (EDX) images were recorded with a JEOL JEM-2100 transmission electron microscope. For sample preparation, a drop of SOM solution dripped onto the copper mesh carbon support film and air-dried. Infrared spectra (IR) were recorded on a Fourier transform infrared spectroscopy (Nicolet 5,700) at room temperature. KBr pressed-disk technique was applied. UV-vis spectra measurements were carried out on a UV-Vis Tu-1901 spectrophotometer using ultrapure water (18.2 MΩ cm) as a blank at 25°C. Cyclic voltammetry (CV) was conducted at ilium XP40 potentiostat with iviumsoft. A homemade three-electrode system was used, with glassy carbon working electrode (0.196 cm^2^), Ag/AgCl reference electrode filled with saturated KCl solution and carbon counter electrode. All the measurements were performed at room temperature in argon atmosphere. 0.1 mM Na_2_SO_4_ was added to the SOM solution to enhance the electrical conductivity.

## Results and Discussion

### Size Control of Soft-Oxometalates Using Surfactant Micelles as Template

**FIGURE 1:**
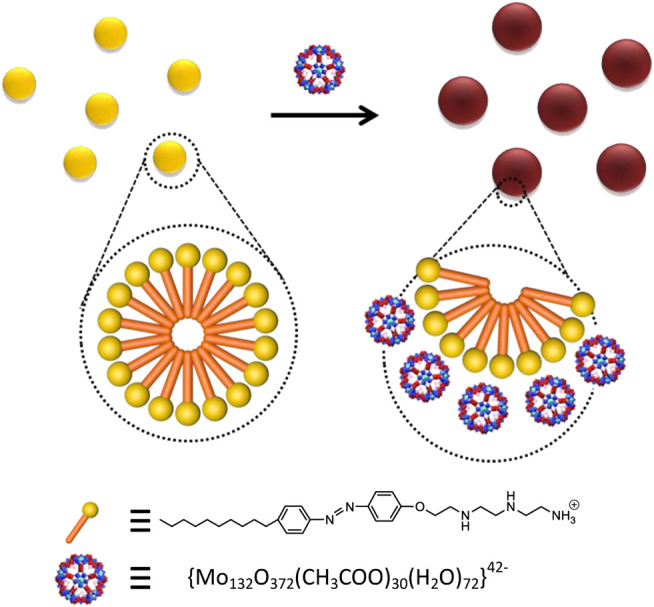
Schematic diagram of the C_10_AZOC_2_N_3_-{Mo_132_} SOM colloids constructed by the electrostatic interaction between the surfactant cationic headgroup and {Mo_132_} Keplerate anion.

Surfactant template method is a simple and effective way to control the morphology and size of nanoparticles. Here, an azobenzene surfactant, C_10_AZOC_2_N_3_ developed in home was used as the template. C_10_AZOC_2_N_3_ molecules self-assembled into spherical micelles in diluted solution with the concentration higher than critical micelle concentration (cmc) (Supplementary Figures S1, S2). There were positive charges on the surface of C_10_AZOC_2_N_3_ micelles because the hydrophilic amino headgroup of the C_10_AZOC_2_N_3_ molecule ionized into a cation in aqueous solution (pH < 11) (Wang et al., 2019). Meanwhile (NH_4_)_42_[Mo^VI^_72_Mo^V^_60_ O_372_COOH)_30_(H_2_O)_72_]·hydrate {Mo_132_} Keplerate anion bore negative charges (Das et al., 2016), which can be adsorbed on the surface of C_10_AZOC_2_N_3_ micelles by electrostatic interaction. Then, C_10_AZOC_2_N_3_-{Mo_132_} SOM colloids were formed by anionic adsorption on the surface of cationic micelles in a non-covalent binding manner (Figure 1).

**FIGURE 2:**
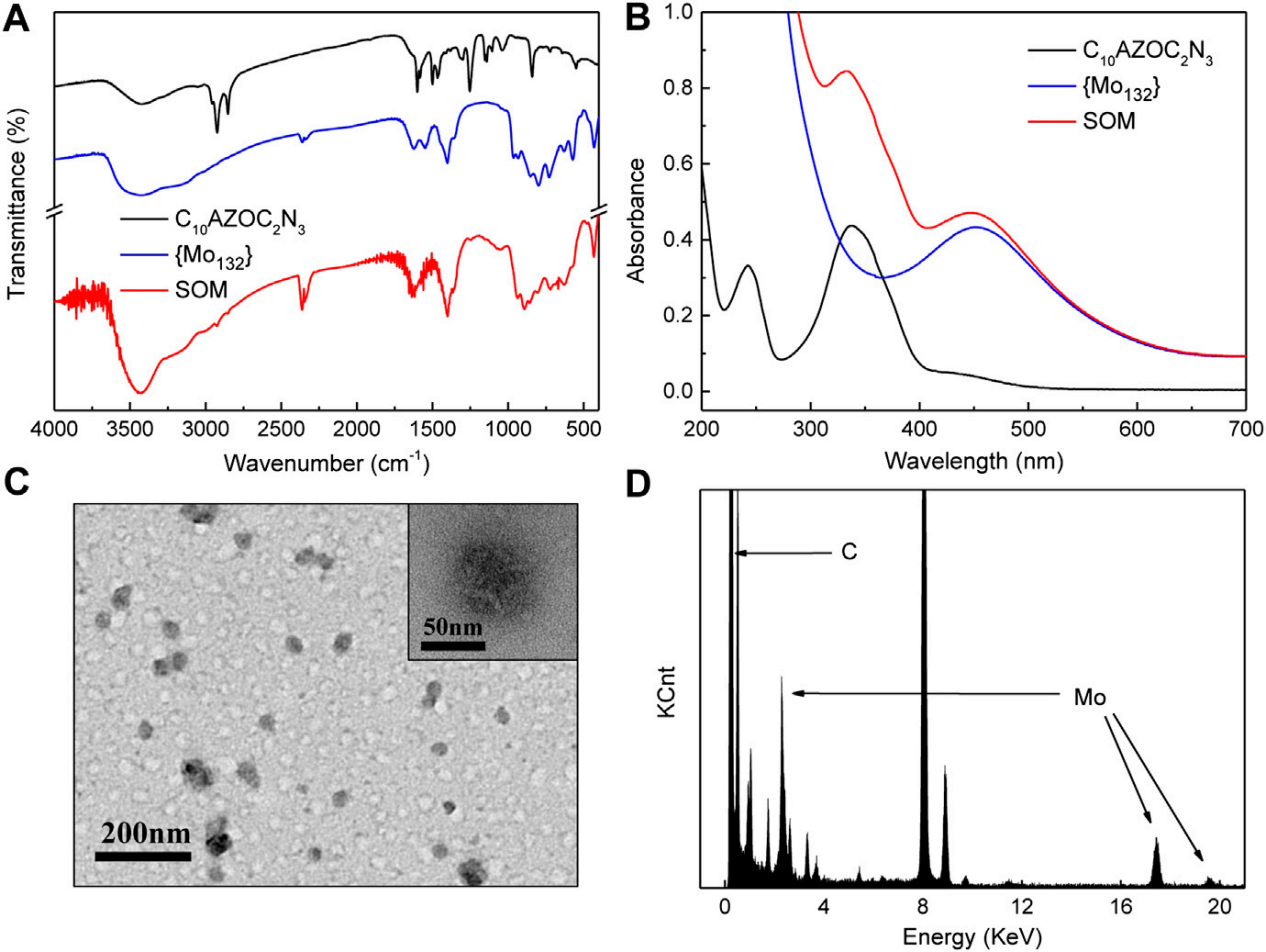
**(A)** IR spectrum of C10AZOC2N3, {Mo132} and SOMs (freeze‐dried). **(B)** UV‐Vis spectra of C10AZOC2N3, {Mo132} and SOMs solution. **(C)** TEM images and **(D)** EDX of SOMs.

The infrared spectra (IR) (Figure 2A) showed the main peaks at 2,923, 2,852 (υ_s Ar-H_), 1,600–1,466 (υ_s Ar_, *δ*_-NH2_, *δ*_-NH-_), 1,253 and 840 (υ_s Ar-N_) cm^−1^ of C_10_AZOC_2_N_3_ and at 1,619 (δ_H2O_), 1,401 (δ_-CH3_, υ_s -COO_, *δ*_as -NH4+_), 964, 729, and 572 cm^−1^ of {Mo_132_} (Das et al., 2016). The ultraviolet-visible (UV-Vis) spectrum (Figure 2B) showed the absorption peak of C_10_AZOC_2_N_3_ at 336 nm and that of {Mo_132_} at 450 nm (Biswas et al., 2016). There was no new absorption peak in IR spectrum and UV-Vis spectra of SOMs, indicating that the main interaction between C_10_AZOC_2_N_3_ and {Mo_132_} was mainly electrostatic interaction. The morphology and element distribution of SOMs were observed by transmission electron microscopy (TEM) and energy dispersive X-ray spectroscopy (EDX) (Figures 2C,D). The TEM images showed that the C_10_AZOC_2_N_3_-{Mo_132_} SOM colloid was spherical and evenly dispersed. EDX showed that Mo was evenly distributed on the colloid, indicating that {Mo_132_} was combined in colloidal particles.

Since the size of SOMs is regulated by adjusting the template micelle size, it is necessary to obtain C_10_AZOC_2_N_3_ micelles that can be customized in size. Regulating the micelle size can be realized by changing solvent polarity, solubilization and so on. Among them, the solubilization of surfactants forms a thermodynamically stable homogeneous system, with the advantage of less surfactant consumption and no obvious change in solvent properties, which is the basis for the application of washing, micellar catalysis, drug delivery and other aspects (Zhao, 2003). When nonpolarized compounds such as saturated alkanes or cycloalkanes are dissolved in surfactant solutions, they are generally added to the hydrophobic core of the micelles, which causes the micelles to become larger. The more nonpolar compounds are added, the larger micelle size is, which is within the upper limit range of solubilization. In this way, the C_10_AZOC_2_N_3_ micelle size can be customized by solubilization, so the SOM size can be regulated, in principle.

**FIGURE 3:**
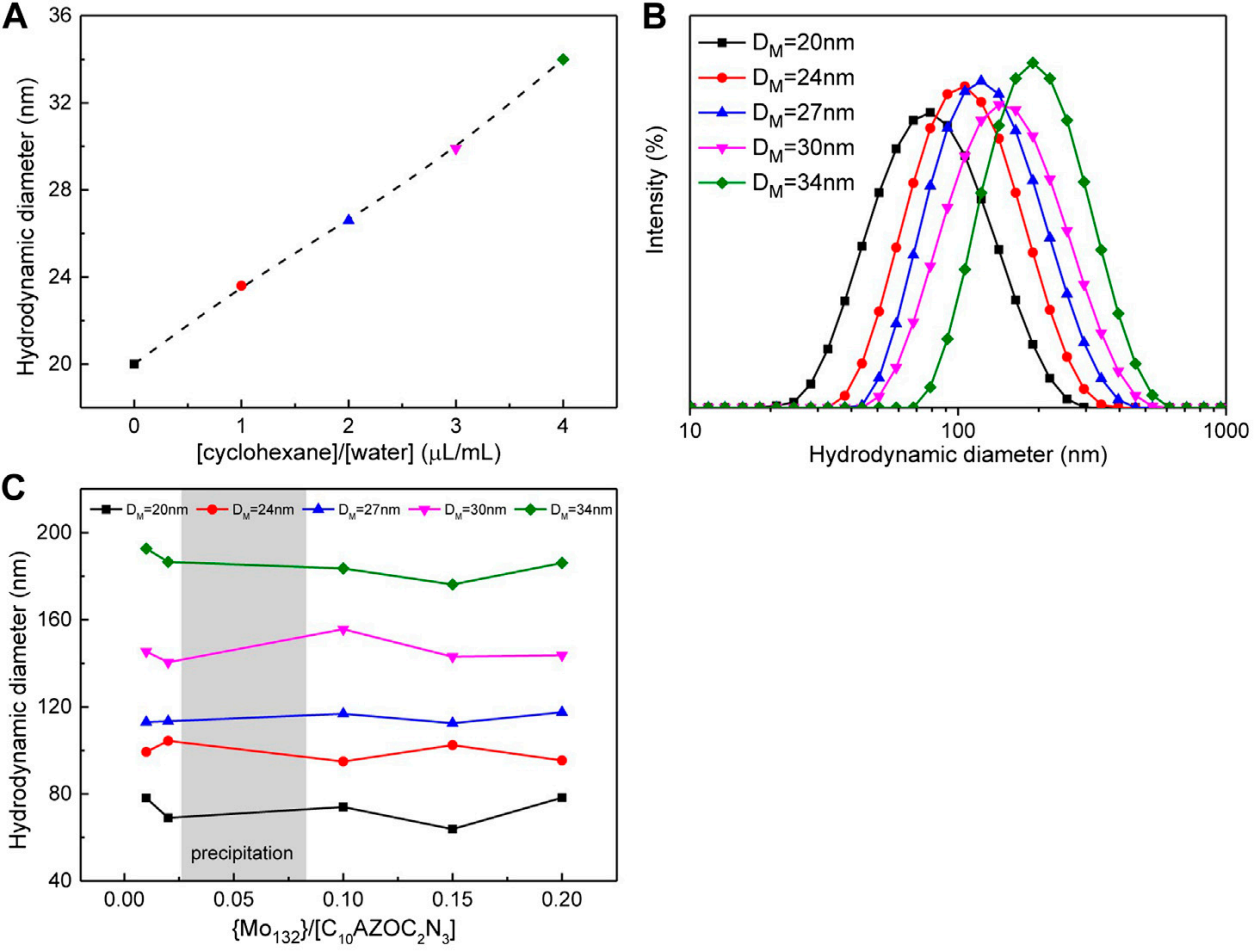
**(A)** Plot of the hydrodynamic diameter of micelles in respect to the volume fraction of cyclohexane, [C10AZOC2N3] = 0.1 mmol/L. **(B)** Hydrodynamic diameter distribution of SOMs templated by five sizes of micelles at [C10AZOC2N3] ‐ 0.1 mmol/L, {Mo132} = 20 μmol/L. **(C)** Hydrodynamic diameter of SOMs templated by five sizes of micelles in different {Mo132}/[C10AZOC2N3].

To obtain different sizes of SOM colloids, we first swelled the surfactant micelles by solubilization of cyclohexane. When the surfactant concentration was fixed at 0.1 mmol/L, the size of micelles increased almost linearly with increasing the volume of cyclohexane within a certain range, usually in a small volume of cyclohexane (Figure 3A). Then, using these micelles as the template, different sizes of the C_10_AZOC_2_N_3_-{Mo_132_} SOM colloids were prepared by adding {Mo_132_}. The hydrodynamic diameter of SOMs were found to increase with increasing the size of micelle templates (Figure 3B), while the size of SOMs prepared with the same micelle size was almost similar, regardless of increase of the concentration ratio of {Mo_132_} and C_10_AZOC_2_N_3_, {Mo_132_}/[C_10_AZOC_2_N_3_] (Figure 3C). These results showed that the size of SOMs can be precisely controlled by controlling the size of micelle templates, and the SOM size only depended on the template size and were not affected by the concentration ratio of {Mo_132_} and surfactant. This may be explained by the self-aggregation of {Mo_132_} itself. When the concentration of {Mo_132_} and C_10_AZOC_2_N_3_ reached a certain ratio, the extra {Mo_132_} Keplerate anions dispersed in solution and self-assembled to form colloids of ten to tens of nanometers with increasing the concentration of {Mo_132_} (Roy et al., 2007).

**FIGURE 4:**
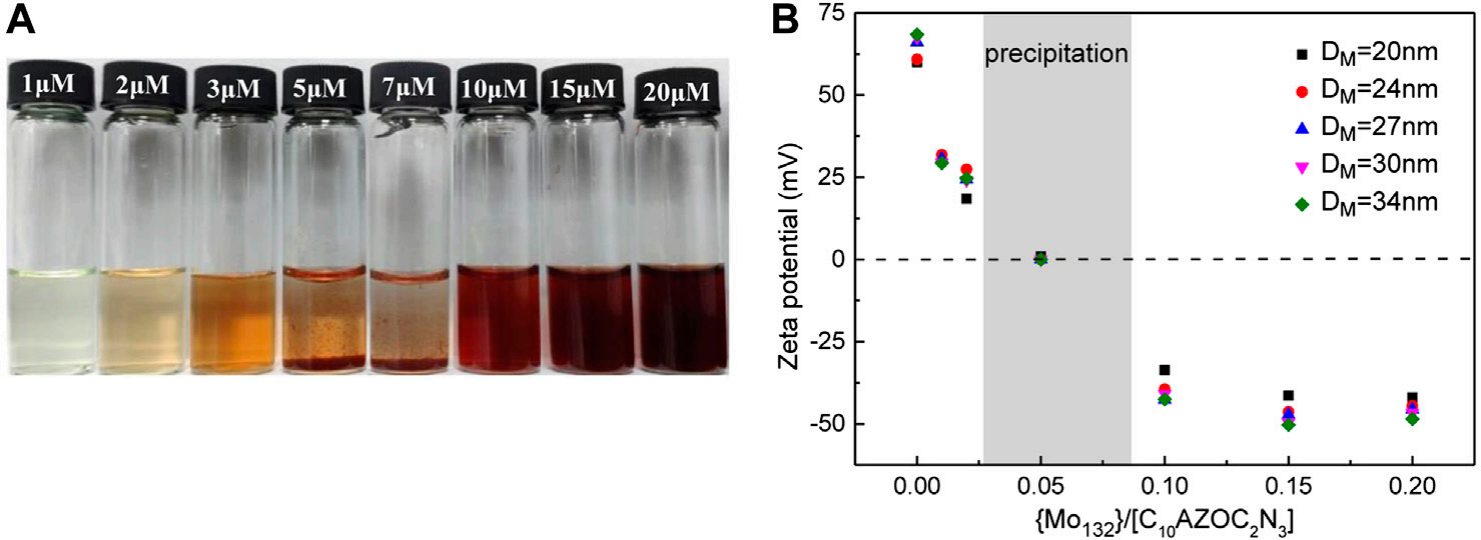
**(A)** Photographs of SOM solutions templated by C10AZOC2N3 micelles in different {Mo132} concentration, [C10AZOC2N3] = 0.1 mmol/L. **(B)** Zeta potentials of C10AZOC2N3 micelles and SOMs.

The phase behavior of the C_10_AZOC_2_N_3_-{Mo_132_} SOM colloids was also observed. With changing the concentration ratio of {Mo_132_} and C_10_AZOC_2_N_3_, SOMs precipitated at the range of {Mo_132_}/[C_10_AZOC_2_N_3_] = 0.04–0.08 (Figure 4A). This can be understood from the point of view that colloidal particles were charged and can be dispersed stably in solution because the electrostatic repulsion between colloidal particles reduced their collision frequency (Wang, 2010). C_10_AZOC_2_N_3_ micelles were stably dispersed in solution with a positively charged surface. Once adding the {Mo_132_}42-, the positive charges were neutralized by these negative charges gradually to zero charge. In that case, there was no charge on the colloidal surface, and then colloidal particles collided with each other and aggregated, showing as precipitation. However, by adding an excess of {Mo132}42, the colloidal surface became negatively charged, and the colloidal particles dispersed stably in solution again (Figure 4A). In the process of gradually increasing the concentration of electrolyte, {Mo_132_}^42-^, typical irregular coagulation (Wang, 2010) such as dispersion, coagulation and dispersion of colloids was exhibited. This mechanism was proved by zeta potential (Figure 4B). When {Mo_132_}/[C_10_AZOC_2_N_3_] was 0, there were only C_10_AZOC_2_N_3_ micelles in solution, and its zeta potential was 60–70 mV, showing a high stability with a high positively charged surface. Then, at low {Mo_132_}/[C_10_AZOC_2_N_3_], the zeta potential went down a lot but it was still positive, meaning there was still positive charge on the colloidal surface. But when {Mo_132_}/[C_10_AZOC_2_N_3_] was 0.04–0.08, the zeta potential went to 0 with a zero charged surface, and the SOM colloids precipitated. Keep increasing {Mo_132_}/[C_10_AZOC_2_N_3_] until higher than 0.1, and the re-stabilization of the SOM dispersion was observed. At this point, the zeta potential was about -40 mV, which meant that the colloidal particles were stabilized by negative charges.

Azobenzene surfactant molecules are known to change conformation from *trans*-isomer to *cis*-isomer under UV light (365 nm) irradiation, resulting in an increase in hydrophilicity and related cmc (Lu et al., 2013; Jia et al., 2015), which endows azobenzene micelles with light responsiveness. Thus, the prepared C_10_AZOC_2_N_3_-{Mo_132_} SOMs may also be photo-responsive. It is then of interest to investigate the effect of light on the morphology of C_10_AZOC_2_N_3_-{Mo_132_} SOMs after UV irradiation.

**FIGURE 5:**
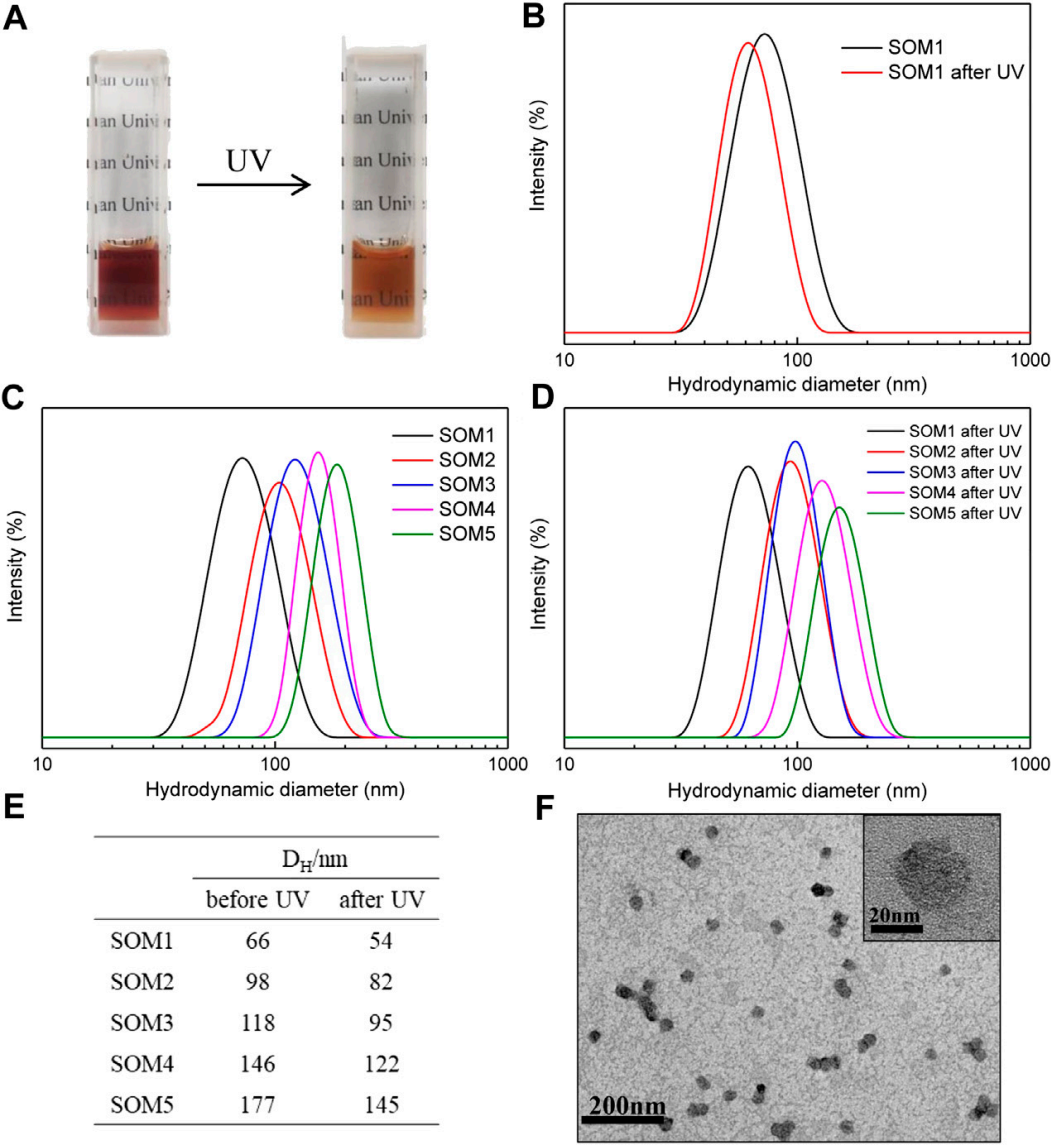
**(A)** Photographs of the C10AZOC2N3‐{Mo132} SOM solutions under UV. **(B ‐E)** the DLS distribution and the hydrodynamic diameters of SOMs in five sizes, SOM1-5, before and after UV irradiation. **(F)** TEM images of SOMs after UV irradiation. {Mo132}/[C10AZOC2N3] = 0.2.

The C_10_AZOC_2_N_3_-{Mo_132_} SOM solutions in all sizes, SOM1-5, were irradiated under UV for 10min, and were found that the solutions changed from dark brown to brown, and their transmittance was increased (Figure 5A). The DLS results showed that SOM1-5 were still uniformly distributed after illumination and their hydrodynamic diameters were generally reduced by 20–30 nm (Figures 5B–E). The reason for the size reduction can be attributed to the fact that C_10_AZOC_2_N_3_ changed from *trans*-isomer to *cis*-isomer under UV irradiation, the molecular hydrophilicity was increased and the micellization ability was weakened. As a result, the micelle aggregation number was decreased and the ability of solubilizing cyclohexane was reduced, leading to the general decrease in the size of micelles (Supplementary Figure S2) and SOMs. The TEM images confirmed that C_10_AZOC_2_N_3_-{Mo_132_} SOMs were still spherical and dispersed colloids after UV irradiation, but their size was smaller than before the illumination (Figure 5F).

### Electrochemical Performance of the Hybrid Surfactant-{Mo_132_} Soft-Oxometalates

Molybdenum polyoxometalates {Mo_132_}, as a photocatalyst, is due to the presence of Mo^V^ and Mo^VI^ centers in the clusters and the intra-valence charge transfer bands in polyoxometalates (Das et al., 2016). Whether the presence of the azobenzene surfactant micelles will cause changes in the redox potential (Mo^V^/Mo^VI^) of {Mo_132_} is of paramount importance for its catalysis ability. In order to find out whether the redox potential of {Mo_132_} clusters is affected by the size control of SOMs by C_10_AZOC_2_N_3_ micelles and light, the cyclic voltammetry (CV) of C_10_AZOC_2_N_3_-{Mo_132_} SOMs before and after UV illumination were characterized.

**FIGURE 6:**
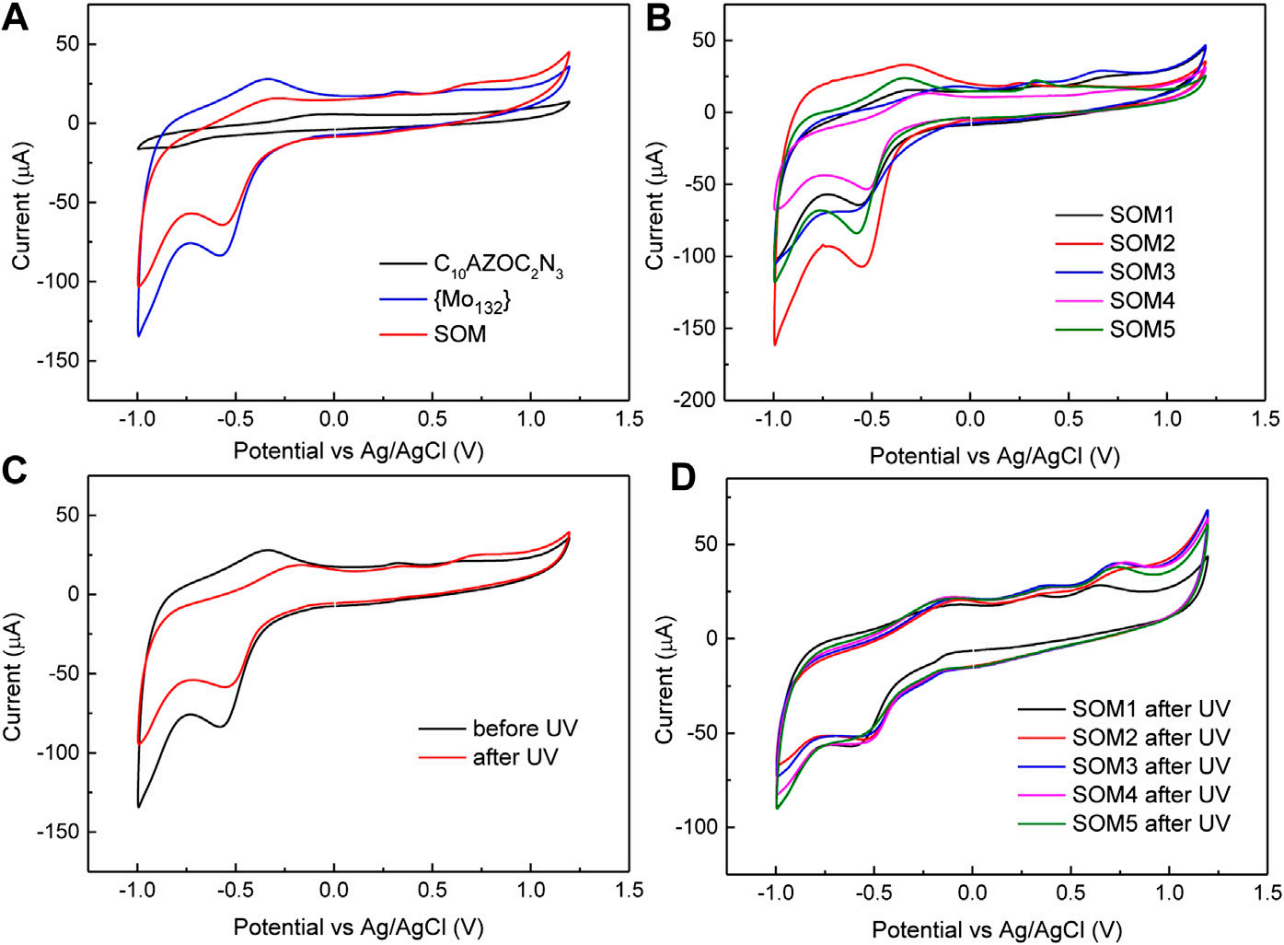
**(A)** Cyclic voltammetry of C10AZOC2N3, {Mo132} and SOMs. **(B)** CV of SOM1‐5 in five sizes. **(C)** CV of {Mo132} before and after UV irradiation. **(D)** CV curves of SOM1‐5 after UV irradiation. [C10AZOC2N3] = 0.1 mmol/L and {Mo132} = 20 μmol/L, scan speed 50 mV/s.

As shown in Figure 6A, pure {Mo_132_} showed a strong reduction peak at −0.55 V and a very weak oxidation peak at −0.45V, attributed to the active redox couple Mo^V^/Mo^VI^. In the presence of surfactants, the current of SOMs was weaker than that of pure {Mo_132_} due to the adsorption of surfactant molecules on the electrode. The strong reduction peak at −0.55 V and corresponding oxidation peak at −0.45 V were clearly observed, indicating that surfactant did not change the redox potential of Mo^V^/Mo^VI^. And what effect does the solubilized cyclohexane inside the SOM colloids have on the redox potential for SOMs of different sizes? CV results (Figure 6B) showed that the redox potential of SOM1-5 of all sizes did not change significantly. We speculated that the interaction and arrangement between {Mo_132_} and surfactant on the surface of SOM colloids of different sizes were almost similar under the condition of the fixed {Mo_132_}/[C_10_AZOC_2_N_3_].

After UV illumination for a certain time, the CV curve of pure {Mo_132_} showed a significant decrease in the current intensity after light exposure, and the oxidation potential was shifted to right from −0.45 V to −0.2 V (Figure 6C), which may be due to the photosensitivity of {Mo_132_} (Lodh et al., 2018). UV light induced unstable dissociation of part of {Mo_132_}, leading to a decrease in the total concentration of {Mo_132_} in solution and a decrease in the current intensity. CV curves of SOM1-5 showed similar changes in current intensity and the redox potential after UV exposure as that of pure {Mo_132_} (Figure 6D). The CV results showed that the size had no significant effect on the redox potential of SOMs, but the redox potential of SOMs was decreased by UV, which was a negative factor. In addition, surfactant did not significantly affect the redox potential of {Mo_132_} clusters, and all changes in the redox potential came from the {Mo_132_} cluster itself. Unlike some reports in the literature that the doping of surfactants made the hybrid POMs show the change of electronic and electric properties to semiconductor characteristics (Klaiber and Polarz, 2016), C10AZOC2N3 micelles were only acted as a controlling template to regulate the SOM morphology and did not participate in the modification of its electrochemical properties in this work.

## Conclusion

The present results show that azobenzene surfactant, C_10_AZOC_2_N_3_, can be used as the template to control the size of {Mo_132_} SOMs. The electrostatic interaction between the positively charged surface of micelles and the negatively charged {Mo_132_} Keplerates made the formation of the hybrid C_10_AZOC_2_N_3_-{Mo_132_} SOMs. The size of SOMs depended on the micelle size that was increased by solubilizing cyclohexane. The concentration ratio of C_10_AZOC_2_N_3_ and {Mo_132_} determined the stability of SOMs. Light-responsive group, azobenzene, in surfactant molecules may provide the *trans*-cis transition under the UV irradiation, but this conformational transition only induced the increase in surfactant hydrophility and poorer micellization ability, resulting in the smaller size of SOMs. Although the redox potential of Mo^V^/Mo^VI^ in {Mo_132_} cluster was not affected in the presence of surfactant, the cyclic voltammetry current was substantially reduced after the UV light irradiation, suggesting that UV light may be a switch in the electrochemical activity of SOMs. In addition, C_10_AZOC_2_N_3_ is pH responsive, and changing the pH of solution may regulate the size of SOMs as well, which needs to be verified by further experiments.

## Data Availability

The raw data supporting the conclusions of this article will be made available by the authors, without undue reservation.
