# Supplementary material for: Fabrication of Soft-Oxometalates {Mo132} Clusters With Novel Azobenzene Surfactants: Size Control by Micelles and Light
Source: Front Chem. 2021 Feb 12;9:625077. doi: 10.3389/fchem.2021.625077 (PMC7928308; doi:10.3389/fchem.2021.625077)
Supplement: Supplementary file 1 [file table1.docx]

Supplementary Material

1. **Synthesis of C_10_AZOC_2_N_3_**

The synthetic route of 1-[2-(4-decylphenylazo-phenoxy)-ethyl]-1-diethylenetriamine (C_10_AZOC_2_N_3_) was shown in Scheme 1. ^1^H and ^13^CNMR spectra were recorded on Bruker Digital NMR Spectrometer Ascend^TM^400 at room temperature and ESI-MS spectra were recorded using LCQ Fleet ion trap mass spectrometer.

**Scheme 1 |** Synthesis pathway for C_10_AZOC_2_N_3_.

## Synthesis of 4-decyl-(4’-hydroxy)azobenzene (C_10_AZOH)

4-Decylaniline (5 g, 21 mmol) was dissolved in the mixture of water (17 mL), hydrochloric acid (6 mL) and acetone (17 mL) under 0°C. Then, sodium nitrite (15 mL, 1.4 mol.L^-1^) was added slowly into this solution. After 30 min stirring under 0º, the aqueous solution (30 mL) of phenol (2 g, 0.021 mol), sodium hydroxide (0.84 g, 0.021 mol) and sodium carbonate (2.3 g, 0.021 mol) was added slowly into this solution, lasting for about 1 h. The mixture was reacted under 0º for 2 h, and then stirred overnight at room temperature. The reaction mixture was filtered. A khaki solid obtained was purified by silica gel column chromatography with petroleum ether and dichloromethane (*v/v,* 1/1) to yield a yellow solid C_10_AZOH (5.8 g, 80%). ^1^H NMR (400 MHz, CDCl_3_) δ (ppm): 0.88 (t, 3H, CH_3_), 1.26 (m, 16H, CH_2_),2.67 (t, 2H, CH_2_), 6.94 (d, 2H, H-Ar), 7.31 (d, 2H, H-Ar), 7.80 (d, 2H, H-Ar), 7.86 (d, 2H, H-Ar).

## Synthesis of 4-decyl-(4’-(2-bromoethyl)phenyl)azobenzene (C_10_AZOC_2_Br)

1,2-dibromoethane (56 g, 0.30mol) and potassium carbonate (5 g, 0.0375mol) was added into ethanol (40 mL) in a 100 mL flask, and heated to 70°C. Then, the acetone solution (100 mL) of C_10_AZOH (5 g, 0.015 mol) was added dropwise under stirring. The solution was refluxed for 12 h. Finally, the solvent was removed and the residual was purified over silica gel using petroleum ether and dichloromethane (*v/v,* 1/1) as the eluent. 5.55 g bright yellow chemical was obtained, yield 84%. ^1^H NMR (400 MHz, CDCl_3_) δ (ppm): 0.88 (t, 3H, CH_3_), 1.26 (m, 16H, CH_2_), 2.67 (t, 2H, CH_2_), 3.69 (t, 2H, CH_2_), 4.38 (t, 2H, CH_2_), 7.01 (d, 2H, H-Ar), 7.31 (d, 2H, H-Ar), 7.79 (d, 2H, H-Ar), 7.89 (d, 2H, H-Ar).

## Synthesis of 1-[2-(4-decylphenylazo-phenoxy)-ethyl]-1-diethylenetriamine (C_10_AZOC_2_N_3_)

C_10_AZOC_2_Br (5 g, 0.011 mol) was dissolved in tetrahydrofuran (100 mL), and added dropwise into the mixture of diethylenetriamine (20 mL) and tetrahydrofuran (20 mL) under stirring at 0°C. Then, the mixture reacted for 12 h at room temperature. The solution was evaporated under vacuum-rotary, and the residue was dissolved in dichloromethane and then extracted with 1 mol/L NaOH and water for 8-10 times, to remove generated hydrogen bromide and excess diethylenetriamine approximately. The oil phase was dried with magnesium sulfate and filtered, and the filtrate was evaporated under vacuum-rotary to obtain the crude product, which was purified by column chromatography with dichloromethane and methyl alcohol (*v/v,* 1/1) to yield a yellow product C_10_AZOC_2_N_3_ (4.72 g, 90%). ^1^H NMR (400 MHz, CDCl_3_) δ (ppm): 0.88 (t, *J* = 6.8 Hz, 3H), 1.26-1.32 (m, 16H), 2.18 (s, 4H), 2.65-2.71 (m, 6H), 2.77-2.85 (m, 4H), 3.06 (t, *J* = 5.2 Hz, 2H), 4.16 (t, *J* = 5.2 Hz, 2H), 7.02 (d, *J* = 9.2 Hz, 2H), 7.31 (d, *J* = 8.4 Hz, 2H), 7.79 (d, *J* = 8.4 Hz, 2H), 7.89 (d, *J* = 9.2 Hz, 2H). ^13^C NMR (133 MHz, CDCl_3_) δ (ppm): 14.14, 22.69, 29.59, 31.35, 35.86, 41.71, 48.68, 49.23, 50.70, 52.39, 53.45, 67.67, 114.67, 122.52, 124.56, 129.06, 145.94, 147.13, 150.95, 161.05. ESI-MS: [M+H]^+^ C_28_H_45_N_5_O^+^, Calcd 468.3, Found 468.3.

## ^1^H, ^13^C NMR and ESI-MS spectra of C_10_AZOC_2_N_3_

**^1^H NMR**

**^13^C NMR**

**ESI-MS**

1. **Surface tension ( γ ) and critical micelle concentration (cmc) of C_10_AZOC_2_N_3_**

**Supplementary Figure 1.** γ−log c plots of C_10_AZOC_2_N_3_ in aqueous solutions at 25 °C, measured by employing the du Noüy ring method (Krüss K100, Germany), and the critical micelle concentration (cmc) is 7.2x10^-6^ mol/L.

1. **Micelles of C_10_AZOC_2_N_3_ in aqueous solution characterized by Dynamic light scattering**

**Supplementary Figure 2.** The scattering intensity versus the hydrodynamic diameters of 0.1mM C_10_AZOC_2_N_3_ aqueous solution before and after UV light irradiation.

1. **UV-vis absorption spectrum of SOMs**

**Supplementary Figure 3.** UV-Vis absorption spectra of SOMs solution at different UV light irradiation conditions, measured on a UV-Vis Tu-1901 spectrophotometer using ultrapure water as a blank at 25 °C.
